# Phenotypic and Genetic Characterization of Flavobacterium psychrophilum Recovered from Diseased Salmonids in China

**DOI:** 10.1128/Spectrum.00330-21

**Published:** 2021-09-15

**Authors:** Shaowu Li, Jingru Chai, Christopher Knupp, Pierre Nicolas, Di Wang, Yongsheng Cao, Furong Deng, Fuguang Chen, Tongyan Lu, Thomas P. Loch

**Affiliations:** a Department of Aquatic Animal Health, Heilongjiang River Fisheries Research Institute, Chinese Academy of Fishery Sciences, Harbin, China; b Key Laboratory of Aquatic Animal Diseases and Immune Technology of Heilongjiang Province, Harbin, China; c College of Fisheries and Life Science, Shanghai Ocean University, Shanghai, China; d Department of Fisheries and Wildlife, College of Agriculture and Natural Resources, Michigan State University, East Lansing, Michigan, USA; e Department of Pathobiology and Diagnostic Investigation, College of Veterinary Medicine, Michigan State University, East Lansing, Michigan, USA; f Université Paris-Saclay, INRAE, MaIAGE, Jouy-en-Josas, France; University of Minnesota

**Keywords:** bacterial coldwater disease, *Flavobacterium psychrophilum*, MLST, genetic diversity, serotype, antimicrobial susceptibility, virulence

## Abstract

Flavobacterium psychrophilum, the etiological agent of bacterial coldwater disease (BCWD) and rainbow trout fry syndrome, causes great economic losses in salmonid aquaculture worldwide. Recent molecular studies have uncovered important epidemiological and ecological aspects of this pathogen; however, such data are lacking for F. psychrophilum populations affecting aquaculture in China. Herein, F. psychrophilum phenotype, genotype, and virulence were characterized for isolates recovered from epizootics in multiple salmonid aquaculture facilities across China. Thirty-one F. psychrophilum isolates, originating from four provinces and three host fish species, were predominantly homogeneous biochemically but represented 5 sequence types (STs) according to multilocus sequence typing (MLST) that belonged to clonal complex CC-ST10 or 3 newly recognized singleton STs. PCR-based serotyping classified 19 and 12 F. psychrophilum isolates into molecular serotypes 1 and 0, respectively, showing an obvious relationship with host species. Antimicrobial susceptibility analysis via broth microdilution revealed reduced susceptibility to enrofloxacin, flumequine, and oxolinic acid, moderate susceptibility to gentamicin, erythromycin, and florfenicol, and variable susceptibility to ampicillin and oxytetracycline. *In vivo* challenge experiments confirmed the ability of two representative Chinese F. psychrophilum isolates to induce typical signs of BCWD and mortality in 1-year-old rainbow trout (Oncorhynchus mykiss). Findings collectively demonstrate (i) that BCWD outbreaks in China studied thus far are caused by F. psychrophilum lineages that are common on other continents (e.g., CC-ST10) and others that have not been reported elsewhere (e.g., ST355, ST356, ST357), (ii) that F. psychrophilum molecular serotypes distinguish isolates from different host fish species, even within STs, and (iii) reduced F. psychrophilum antimicrobial susceptibility against compounds used for BCWD control in China.

**IMPORTANCE** Flavobacterium psychrophilum causes substantial economic losses in salmonid aquaculture worldwide. Although this bacterium is also believed to be a disease source in China, published reports of its presence do not yet exist. Herein, F. psychrophilum was linked to multiple disease outbreaks in several salmonid aquaculture facilities within four Chinese provinces, and polyphasic characterization revealed that most isolates were genetically distinct from strains recovered on other continents. Analyses further revealed the predominating molecular serotypes, antimicrobial susceptibility profiles, and pathogenic potential of two representative recovered isolates. Collectively, the results presented here provide important data on the epidemiology and disease ecology of F. psychrophilum in China and pave the way for targeted prevention and control methods to be pursued in the future.

## INTRODUCTION

Flavobacterium psychrophilum, the etiological agent of bacterial coldwater disease (BCWD) and rainbow trout fry syndrome, is considered one of the most important and damaging bacterial pathogens in freshwater salmonid aquaculture (1–3). This pathogen affects fish at all life stages; however, fry and fingerlings are considered most susceptible to F. psychrophilum-induced disease outbreaks. Disease usually occurs at water temperatures ranging from 3°C to 15°C, and almost all species of salmonid fish are considered susceptible; however, coho salmon (Oncorhynchus kisutch), rainbow trout (Oncorhynchus mykiss), and ayu (Plecoglossus altivelis) are particularly vulnerable to this disease (4, 5).

Originally isolated from diseased coho salmon in the United States in the 1940s (6), F. psychrophilum is now recognized as a salmonid pathogen of global significance, having been recovered from diseased fish in Oceania, Europe, North America, South America, and Asia (3). The vertical transmission of F. psychrophilum from infected broodstock to offspring via reproductive fluids and infected eggs, coupled with the national and international trade of live salmonids and their eggs, has been hypothesized as an important factor in the apparent global spread of this bacterium (7, 8). More recent studies investigating the molecular epidemiology of F. psychrophilum have supported this hypothesis, particularly those utilizing multilocus sequence typing (MLST) analysis (9, 10). Additionally, such studies have highlighted the predominating F. psychrophilum MLST clonal complexes (CCs) and sequence types (STs) responsible for disease outbreaks in multiple countries (9–13) and also suggested that some F. psychrophilum genetic variants have an apparent proclivity for certain salmonid host species (10, 12, 13). Moreover, previous studies also suggested a relationship between some F. psychrophilum MLST genetic variants and reduced *in vitro* susceptibility to certain antimicrobial compounds, a concern for pathogen control purposes in aquaculture given the lack of efficacious preventative measures and obstacles in developing protective vaccination strategies against F. psychrophilum (14–16).

Serological diversity among F. psychrophilum isolates has also been studied, resulting in several different serotyping schemes, such as serovar-1 to serovar-7, Fd/Th/Fp^T^, and others (17, 18). A relationship between serotype and host species has been found for some variants. For example, serotypes O-1, O-2, and O-3 were reported to correspond to isolates infecting coho salmon, ayu, and rainbow trout, respectively (5). More recently, a PCR-based method was developed for the determination of previously described F. psychrophilum serotypes, increasing the reproducibility of “serotyping” data (19). Subsequent studies using this molecular serotyping scheme have provided further evidence for various degrees of host-molecular serotype associations (20, 21).

Despite the fact that total salmonid production in China is currently about 40,000 tons per year (22), with production efforts intensifying, the characteristics of F. psychrophilum lineages found in China in general, and any association with virulence, antimicrobial susceptibility, and disease outbreaks in aquaculture in particular, have not yet been thoroughly investigated. The aforementioned gaps in knowledge are of growing concern given that bacterial disease outbreaks are becoming more prevalent and F. psychrophilum is increasingly being recognized as a major contributor to disease-associated losses, ranging from 10 to 60% in affected artificially reared salmonid stocks (23). With a long-term goal of developing improved disease prevention and control methods for Chinese aquaculture, the current study was undertaken to phenotypically and molecularly characterize multiple F. psychrophilum isolates recovered from disease outbreaks in several Chinese aquaculture facilities, investigate their virulence under controlled laboratory conditions, and evaluate their *in vitro* antimicrobial susceptibility using broth microdilution.

## RESULTS

### Fish clinical signs and sampling.

**FIG 1:**
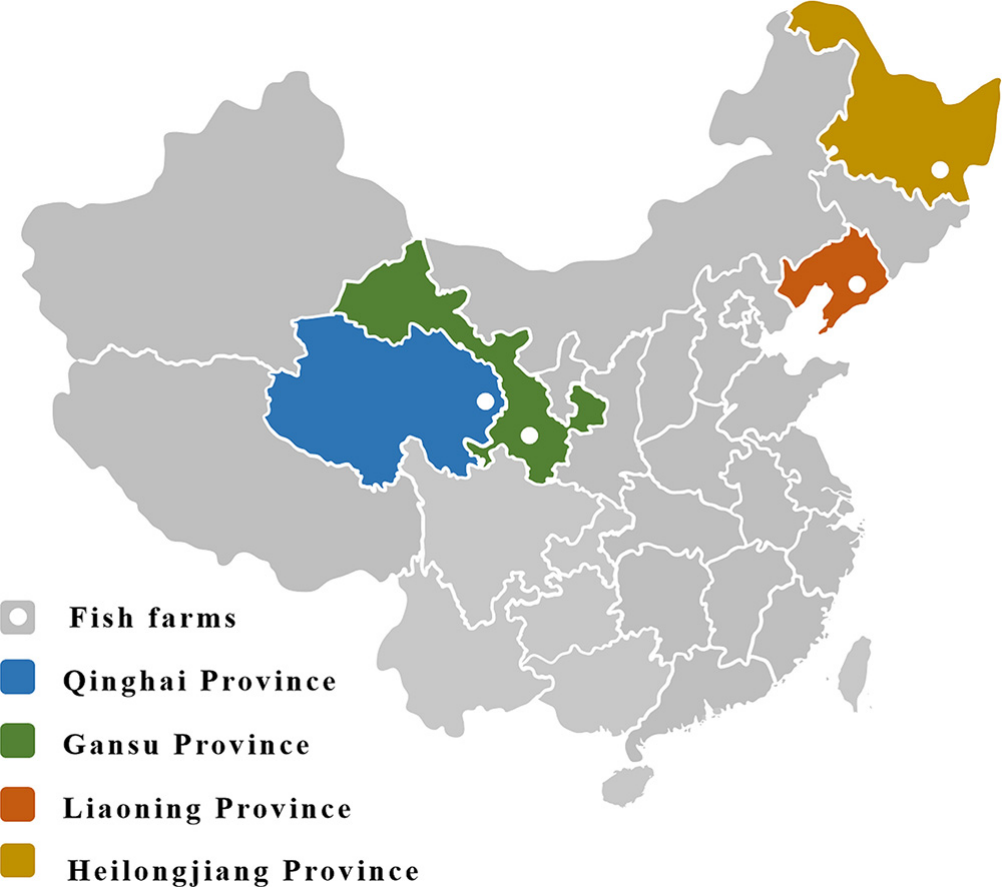
Map of China. The four provinces of China where fish were recovered from in this study are colored, and the specific location of the fish farm is indicated with a white circle.

From 2016 to 2019, diseased rainbow trout, brook trout (Salvelinus fontinalis), and masou salmon (Oncorhynchus masou masou) were collected from four aquaculture facilities within the Liaoning, Qinghai, Gansu, or Heilongjiang provinces (Fig. 1). Collectively, these four provinces are responsible for more than 50% of the annual salmonid production in China.

**FIG 2:**
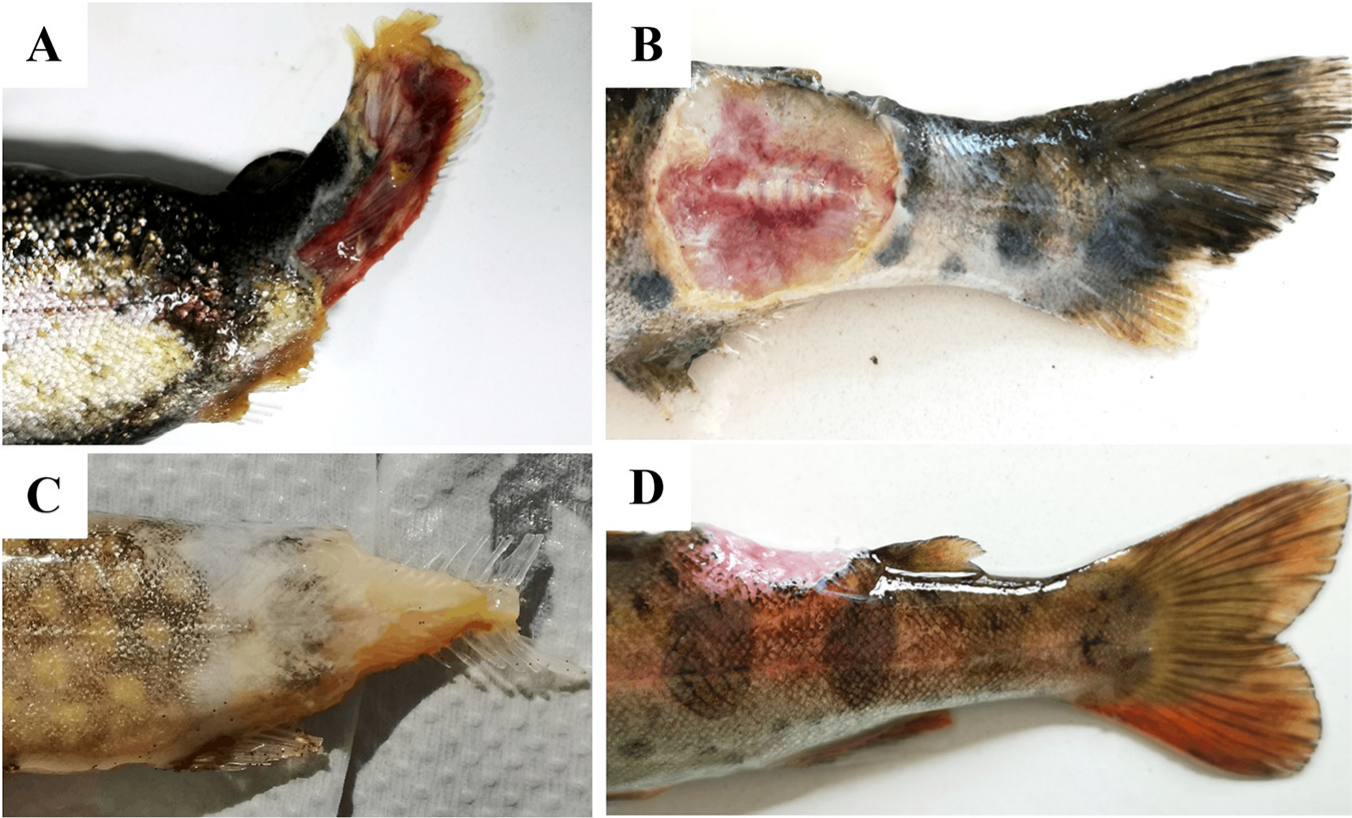
Clinical signs of naturally diseased salmonids from which Flavobacterium psychrophilum was recovered. (A) Diseased rainbow trout (Oncorhynchus mykiss) showing severe caudal fin erosion and a deep, focally extensive hemorrhagic ulceration of the caudal peduncle. (B) Diseased masou salmon (*O. masou masou*) with a deep, focally extensive and hemorrhagic ulceration near the caudal peduncle. (C) Diseased brook trout (*Salvelinus fontinalis*) with severe focally extensive to diffuse ulceration of the caudal peduncle, along with near complete erosion of the caudal fin. (D) Diseased masou salmon with a deep focal ulceration on the dorsal aspect of the fish.

Cumulative mortality in groups of diseased salmonids that were sampled ranged from 10 to 30% over a period of 2 to 3 months. Diseased fish exhibited typical signs of BCWD (Fig. 2), including deep and focally extensive hemorrhagic ulceration of the caudal peduncle or on the dorsal aspect and/or complete erosion of the caudal fin. In severely affected fish, gross signs consistent with saprolegniasis were observed, especially at lower water temperature. Noteworthy gross internal findings included liver pallor, splenomegaly, and severe enteritis. A total of 88 diseased fish, representative of three fish species, were sampled for bacterial isolation (Table 1).

**TABLE 1:** Summary information for the 31 recovered Chinese Flavobacterium psychrophilum isolates, including fish species, collection sites, sampling date, and number of juvenile fish suffering from disease outbreaks in fish farms

Fish species	Collection site	Sampling date	No. of sampled fish^*a*^	No. of recovered F. psychrophilum isolates
*O. mykiss*	Liaoning	October 2016	3	1
		June 2017	15	4
*O. mykiss*	Gansu	July 2016	6	1
		May 2017	5	1
*O. mykiss*	Qinghai	June 2018	6	1
*O. mykiss*	Heilongjiang	October 2019	3	2
*O. masou masou*	Heilongjiang	October 2019	27	8
*O. masou masou*	Heilongjiang	December 2019	5	3
*S. fontinalis*	Heilongjiang	December 2019	18	10
				
Total			88	31

a All the samples were collected from diseased fish.

### Bacterial isolation, identification, and phenotypic characterization.

**FIG 3:**
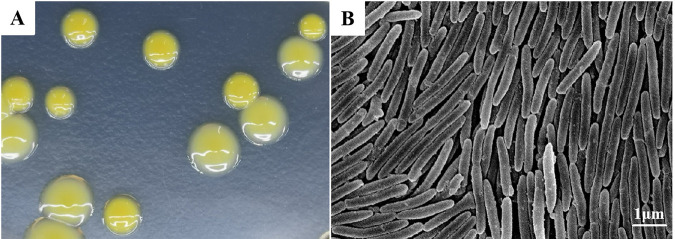
Morphology observation of Flavobacterium psychrophilum isolate CN06. (A). Colony morphology of a representative F. psychrophilum isolate recovered from a Chinese aquaculture facility on a TYES plate. (B) F. psychrophilum colony comprised of slender rods as viewed by scanning electron microscopy (SEM, SU8010, ×10,000).

Thirty-one bacterial isolates were recovered from multiple salmonid disease outbreaks (Table 1): 10 from brook trout, 10 from rainbow trout, and 11 from masou salmon. All isolates produced colonies of a morphology consistent with that of F. psychrophilum, in that they were yellow, circular, and convex, with a thin spreading edge (Fig. 3A). Actively growing cells of one isolate taken as representative (CN06) were approximately 0.26 μm in diameter and between 1.53 and 2.77 μm in length (Fig. 3B). All 31 isolates were catalase and cytochrome oxidase positive, were esculin negative, produced a flexirubin-type pigment, and hydrolyzed tyrosine but not starch. Moreover, none of the 31 tested isolates utilized glucose, fructose, galactose, mannose, or glycerol, nor did they produce H_2_S or grow at 30°C or in the presence of 1.5% NaCl. Variation in isolate phenotype was occasionally noted, whereby 21/31 isolates showed gliding motility, 28/31 hydrolyzed gelatin, and 2/31 grew in tryptic soy broth (TSB).

Sequencing of the 16S rRNA gene confirmed the identification of the 31 yellow-pigmented bacteria as F. psychrophilum, as evidenced by high percent similarity (99.13 to 99.57%) to the F. psychrophilum reference strain (IFO 15942) using EZBio Cloud database. The 16S rRNA gene sequences of the 31 F. psychrophilum isolates have been deposited with accession numbers MN653377 to MN653384 and MT249231 to MT249253 into GenBank of the NCBI.

### Genetic characterization.

MLST analysis of the 31 F. psychrophilum Chinese isolates recovered in this study identified five STs defined as distinct combinations of allele types (ATs) at the seven loci of the MLST scheme (Table S1): ST356 (12 isolates), ST357 (9 isolates), ST12 (7 isolates), ST355 (2 isolates), and ST78 (1 isolate). Across the 5,799-bp alignment resulting from the concatenation of the seven loci, the average pairwise nucleotide divergence between the five STs ranged from 0.0007 bp^−1^ to 0.0066 bp^−1^ (from 99.93% to 99.34% similarity). Details on STs and ATs are given in Table S1 and Table 2. Sequences and background information on isolates were deposited in PubMLST (https://pubmlst.org/fpsychrophilum/) along with 37 F. psychrophilum new isolates from the United States (Table S2).

**TABLE 2:** Complete information of the 31 Chinese Flavobacterium psychrophilum isolates presented in this study, including year of isolation, location of isolation, host species, tissue of isolation, host life stage, genotype, and serotype. Data are arranged by clonal complex (CC) and then sequence type (ST)

Isolate ID	Yr of isolation	Location of isolation	Species	Isolation tissue	Fish weight (g)	ST	CC	Serotype
CN01	2017	Liaoning	*O. mykiss*	Kidney	30.5	ST12	CC-ST10	1
CN02	2017	Liaoning	*O. mykiss*	Skin ulcer	25.6	ST12	CC-ST10	1
CN03	2017	Liaoning	*O. mykiss*	Skin ulcer	28.2	ST12	CC-ST10	1
CN04	2016	Liaoning	*O. mykiss*	Kidney	18.3	ST12	CC-ST10	1
CN05	2016	Gansu	*O. mykiss*	Kidney	22.5	ST12	CC-ST10	1
CN06	2017	Gansu	*O. mykiss*	Skin ulcer	24.1	ST12	CC-ST10	1
CN08	2018	Qinghai	*O. mykiss*	Spleen	12.7	ST12	CC-ST10	1
CN07	2017	Liaoning	*O. mykiss*	Skin ulcer	10.1	ST78	CC-ST10	1
CN11	2019	Heilongjiang	*O. mykiss*	Spleen	10.5	ST355^*a*^	Singleton	1
CN12	2019	Heilongjiang	*O. mykiss*	Spleen	42.4	ST355^*a*^	Singleton	1
CN09	2019	Heilongjiang	*O. masou masou*	Kidney	8.2	ST356^*a*^	Singleton	0
CN10	2019	Heilongjiang	*O. masou masou*	Kidney	5.8	ST356^*a*^	Singleton	0
CN13	2019	Heilongjiang	*O. masou masou*	Kidney	10.3	ST356^*a*^	Singleton	0
CN14	2019	Heilongjiang	*O. masou masou*	Kidney	9.2	ST356^*a*^	Singleton	0
CN15	2019	Heilongjiang	*O. masou masou*	Kidney	9.0	ST356^*a*^	Singleton	0
CN16	2019	Heilongjiang	*O. masou masou*	Kidney	7.9	ST356^*a*^	Singleton	0
CN17	2019	Heilongjiang	*O. masou masou*	Kidney	11.3	ST356^*a*^	Singleton	0
CN20	2019	Heilongjiang	*O. masou masou*	Kidney	10.5	ST356^*a*^	Singleton	0
CN19	2019	Heilongjiang	*S. fontinalis*	Skin ulcer	16.2	ST356^*a*^	Singleton	1
CN25	2019	Heilongjiang	*S. fontinalis*	Kidney	13.6	ST356^*a*^	Singleton	0
CN30	2019	Heilongjiang	*S. fontinalis*	Skin ulcer	14.4	ST356^*a*^	Singleton	1
CN31	2019	Heilongjiang	*S. fontinalis*	Skin ulcer	15.2	ST356^*a*^	Singleton	1
CN18	2019	Heilongjiang	*O. masou masou*	Kidney	8.8	ST357^*a*^	Singleton	0
CN21	2019	Heilongjiang	*O. masou masou*	Kidney	15.3	ST357^*a*^	Singleton	0
CN22	2019	Heilongjiang	*O. masou masou*	Kidney	10.2	ST357^*a*^	Singleton	0
CN23	2019	Heilongjiang	*S. fontinalis*	Kidney	21.2	ST357^*a*^	Singleton	1
CN24	2019	Heilongjiang	*S. fontinalis*	Skin ulcer	19.5	ST357^*a*^	Singleton	1
CN26	2019	Heilongjiang	*S. fontinalis*	Skin ulcer	33.8	ST357^*a*^	Singleton	1
CN27	2019	Heilongjiang	*S. fontinalis*	Skin ulcer	26.2	ST357^*a*^	Singleton	1
CN28	2019	Heilongjiang	*S. fontinalis*	Skin ulcer	25.4	ST357^*a*^	Singleton	1
CN29	2019	Heilongjiang	*S. fontinalis*	Skin ulcer	18.1	ST357^*a*^	Singleton	1

a STs that were newly discovered in this study.

**FIG 4:**
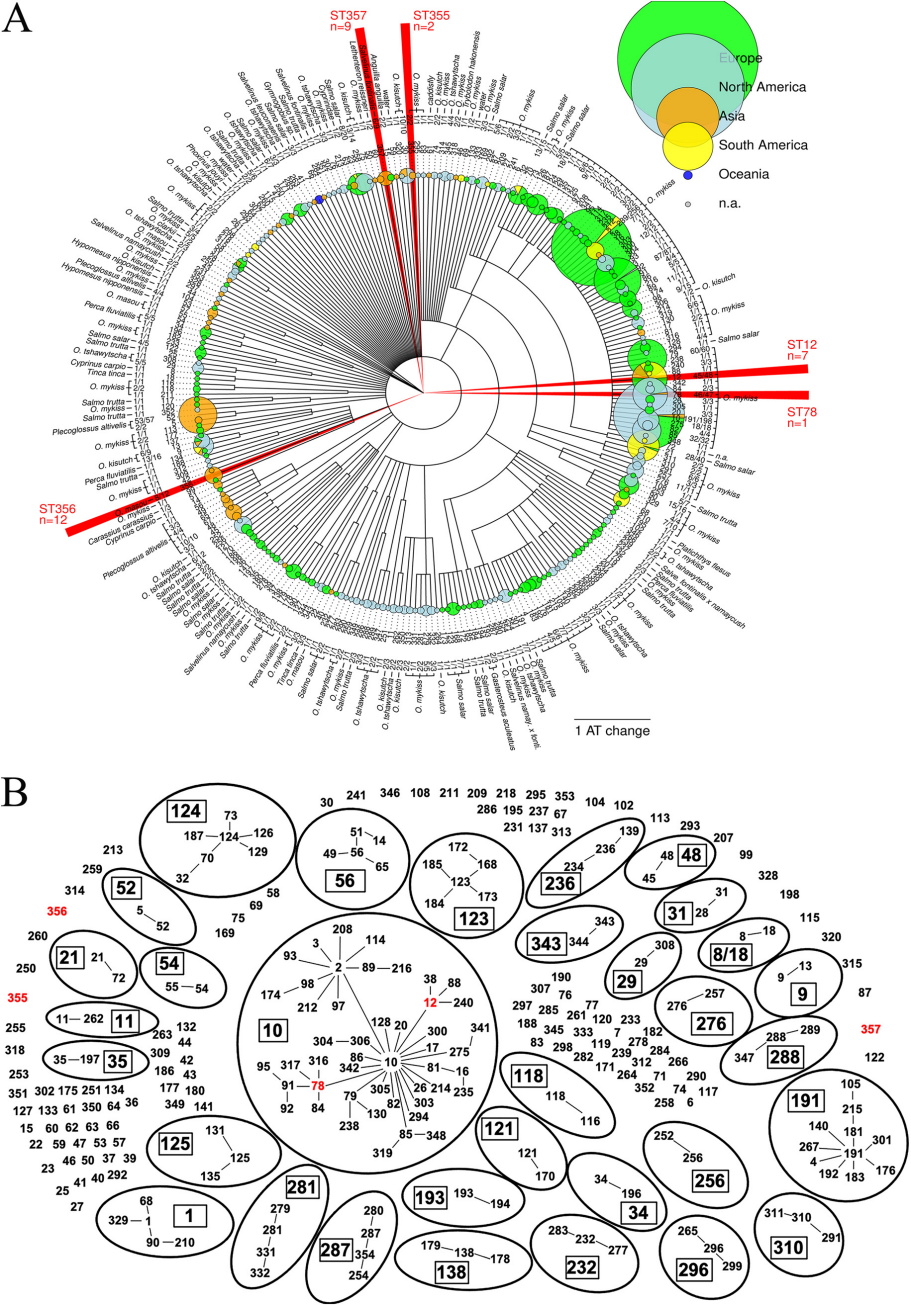
(A) Single-linkage dendrogram of 1,544 Flavobacterium psychrophilum isolates (263 STs) genotyped using MLST, worldwide. Each tip corresponds to one ST. Information reported for each ST on the periphery of the fan representation is, in outward direction, geographical origin (pie chart whose area is proportional to the number of isolates from each continent), ST, number of isolates from a given fish host, fish host. STs recovered in China are highlighted in red. (B) goeBURST diagram of the same data set. STs in red were recovered in China. Clonal complexes (CCs), defined as groups of isolates connected by single-locus variant (SLV) links, are indicated by ellipses. A CC is named after the most likely founding ST identified as the ST with the highest number of SLVs. If multiple STs have the same number of SLVs, the founder is named after the ST with the most isolates. If both STs have the same number of isolates, the CC is named after the earliest found ST. The founding ST of a CC is enclosed within a box. Only SLV links corresponding to this reconstructed history of the CC are represented.

To understand the genetic relationships of the recovered F. psychrophilum isolates with one another and with worldwide diversity, we performed a global comparison of STs and AT profiles between 1,544 isolates (263 STs) resulting from this study and previously published studies. The results are shown in Figure 4A, where information on geographical origin and fish host is superimposed on a hierarchical clustering tree displaying relationships between STs. A goeBURST diagram highlighting relationships within clonal complexes is also presented Figure 4B.

The 8 isolates retrieved from Liaoning, Qinghai, and Gansu provinces (all isolated from rainbow trout) were identified as ST12 and ST78, belonging to one clonal complex (CC-ST10, see Fig. 4B). In contrast, the three STs found in Heilongjiang province (ST355, ST356, and ST357) are new, and their closest matches in the database of worldwide diversity differ by 4 ATs for ST356 and by 5 ATs for ST355 and ST357 (Fig. 4A; Table S1), which can also be analyzed in PubMLST (https://pubmlst.org/fpsychrophilum/). Three fish hosts are represented, and some degree of statistical association exists between ST and fish host for these three isolates (*P* = 0.0045 for the Fisher test on 3 by 3 matrix). In detail, ST355 accounts for the 2 isolates from rainbow trout, while ST356 and ST357 account for isolates from brook trout (4 ST356 and 6 ST357) and masou salmon (8 ST356 and 3 ST357).

The mPCR serotyping scheme assigned 19 F. psychrophilum isolates as type 1 and 12 isolates as type 0 (Table 2). There was a strong correlation between serotype and host fish species. For example, all strains recovered from masou salmon belonged to type 0 (11/11), whereas all strains retrieved from rainbow trout belonged to type 1 (10/10). Among the 10 isolates recovered from brook trout, 9 belonged to type 1, whereas the remaining isolate belonged to type 0. Among the 9 F. psychrophilum ST357 isolates, 3 recovered from masou salmon belonged to type 0, whereas the 6 recovered from brook trout belonged to type 1 (Table 2).

### Virulence characterization.

**FIG 5:**
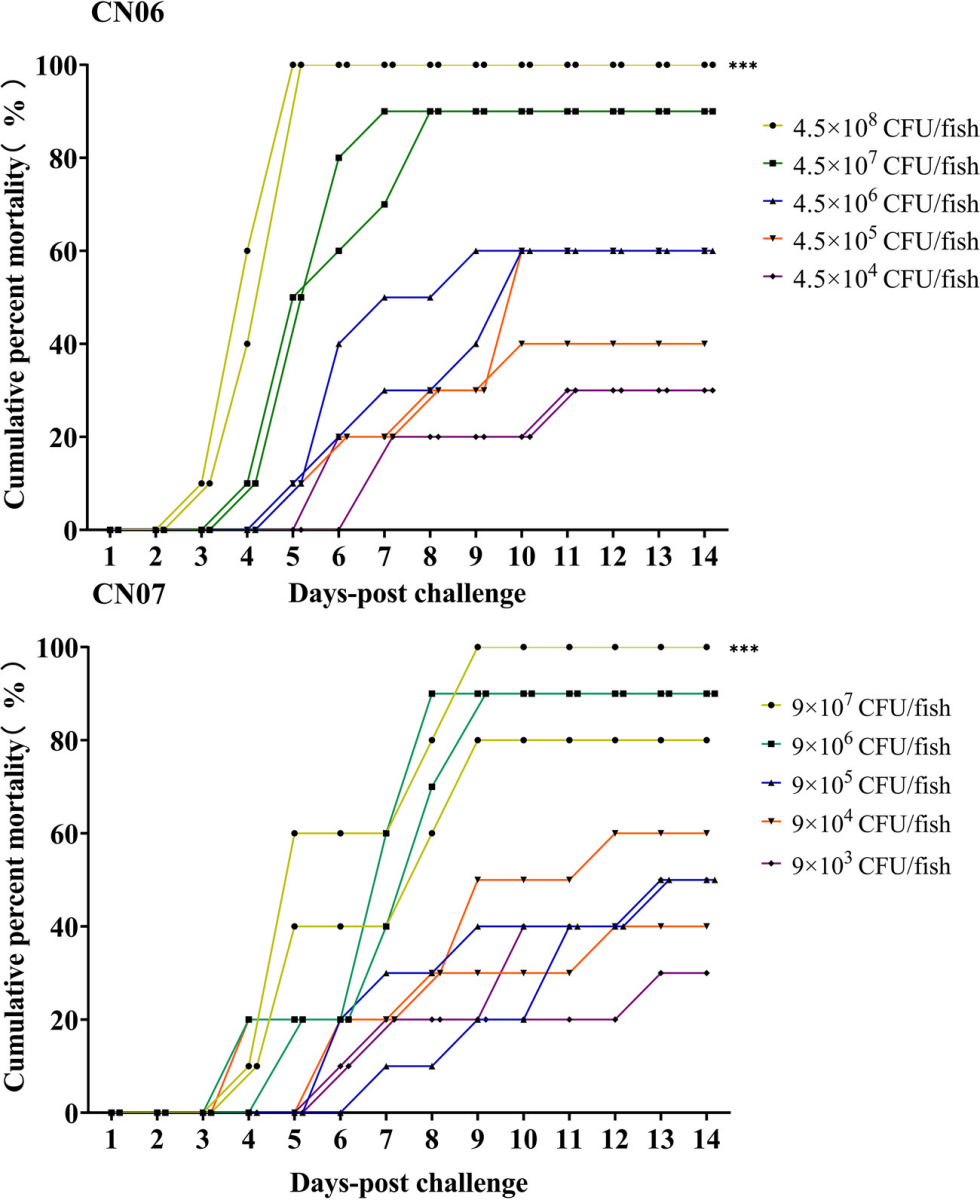
Cumulative percent mortality in juvenile rainbow trout (Oncorhynchus mykiss) experimentally challenged with two Chinese Flavobacterium psychrophilum strains (CN06 and CN07).

Two F. psychrophilum isolates (CN06 and CN07), representative of ST12 and ST78 within CC-ST10, induced gross signs of BCWD and caused mortality in rainbow trout when intramuscularly injected at a range of doses (Fig. 5). Observed external clinical signs of BCWD included skin ulceration, diffuse melanosis, fin erosion, and erratic swimming, whereas internal BCWD signs included liver and heart pallor, splenomegaly, kidney congestion, and severe enteritis. Cumulative mortality ranged from 30 to 100% when fish were challenged with CN06 and 40 to 90% when they were challenged with CN07 (Fig. 5). Based on these results, the 50% lethal dose (LD_50_) for CN06 (ST12) and CN07 (ST78) was estimated to be 7.1 × 10^5^ CFU and 1.1 × 10^5^ CFU, respectively.

F. psychrophilum was reisolated from the muscle, spleen, kidney, and liver of all moribund and dead experimental fish but was never isolated from any of the negative-control fish. According to the F. psychrophilum-specific qPCR assay results, the tissues (including muscle, kidney, spleen, and liver) were also positive to be infected by F. psychrophilum (Table 3).

**TABLE 3:** Bacterial loads in different tissues of rainbow trout (Oncorhynchus mykiss) at 24 h postinfection with Flavobacterium psychrophilum CN06 and CN07 isolates through qPCR assay

Isolate	Tissue	Ct values	Bacterial load (copies/μl)
CN06	Spleen	26.72 ± 0.59	(1.26 ± 0.37) × 10^4^
	Liver	32.29 ± 0.05	(5.40 ± 0.16) × 10^2^
	Kidney	30.54 ± 0.63	(1.49 ± 0.46) × 10^3^
	Muscle	19.66 ± 0.56	(6.48 ± 2.07) × 10^5^
CN07	Spleen	30.34 ± 0.30	(1.62 ± 0.28) × 10^3^
	Liver	32.73 ± 1.07	(4.70 ± 2.37) × 10^2^
	Kidney	30.73 ± 0.34	(1.30 ± 0.23) × 10^3^
	Muscle	21.71 ± 0.08	(2.00 ± 0.85) × 10^5^

### Antimicrobial susceptibility profiles.

The inoculum suspensions for the MIC tests contained 5 × 10^5^ CFU/ml, as determined by colony counts, and the MIC values produced by the two quality control strains grown at 18°C were within accepted CLSI ranges (Table 4).

**TABLE 4:** Summary of antimicrobial susceptibility profiles for 31 Flavobacterium psychrophilum isolates recovered in China^*a*^

Strains used in this study	MIC value (μg/ml) (R/M/S) of drug:							
	AMP	ENRO	ERY	FFN	FLUQ	GEN	OXO	OXY
ATCC 25922	2–8		4–16	4–32	0.06–0.25	0.25–1	0.03–0.12	0.12–1
ATCC 33658	0.06–0.25	0.004–0.03	4–16	0.25–1	0.015–0.06	0.25–1	0.008–0.03	0.06–0.25
CN01^*b*^	<0.001 (S)	0.0625 (R)	0.5 (M)	4 (R)	2 (R)	0.5 (M)	0.25 (M)	4 (M)
CN02^*b*^	<0.001 (S)	0.03 (M)	0.3 (S)	0.5 (M)	2 (R)	0.5 (M)	0.5 (R)	4 (M)
CN03^*b*^	<0.001 (S)	0.5 (R)	0.5 (M)	2 (R)	1 (R)	0.25 (M)	0.25 (M)	2 (M)
CN04^*b*^	<0.001 (S)	0.0625 (R)	0.5 (M)	1 (M)	2 (R)	1 (M)	0.5 (R)	16 (R)
CN07^*b*^	<0.001 (S)	0.03 (M)	0.125 (S)	0.5 (M)	4 (R)	0.5 (M)	4 (R)	0.0526 (M)
CN05^*c*^	<0.001 (S)	1 (R)	0.5 (M)	1 (M)	4 (R)	0.5 (M)	0.5 (R)	8 (M)
CN06^*c*^	<0.001 (S)	0.5 (R)	0.5 (M)	8 (R)	4 (R)	0.0625 (M)	2 (R)	4 (M)
CN08^*d*^	<0.001 (S)	0.5 (R)	8 (R)	2 (R)	4 (R)	0.5 (M)	0.5 (R)	4 (M)
CN09^*e*^	0.125 (R)	>0.5 (R)	0.5 (M)	0.5 (M)	8 (R)	0.25 (M)	8 (R)	0.015 (S)
CN10^*e*^	0.125 (R)	>0.5 (R)	0.5 (M)	0.5 (M)	8 (R)	0.125 (M)	8 (R)	0.015 (S)
CN11^*e*^	0.0625 (M)	0.015 (M)	2 (M)	1 (M)	0.125 (M)	0.25 (M)	0.125 (M)	0.0625 (M)
CN12^*e*^	0.125 (R)	0.004 (M)	1 (M)	0.5 (M)	0.0312 (M)	0.25 (M)	0.125 (M)	0.015 (S)
CN13^*e*^	0.125 (R)	>0.5 (R)	0.25 (S)	0.5 (M)	4 (R)	0.125 (M)	8 (R)	<0.015 (S)
CN14^*e*^	0.125 (R)	0.5 (R)	0.5 (M)	0.5 (M)	4 (R)	0.25 (M)	8 (R)	0.03 (M)
CN15^*e*^	0.125 (R)	0.0625 (R)	1 (M)	1 (M)	1 (R)	0.25 (M)	2 (R)	0.015 (S)
CN16^*e*^	0.125 (R)	0.0625 (R)	0.5 (M)	0.5 (M)	1 (R)	0.125 (M)	2 (R)	0.015 (S)
CN17^*e*^	0.125 (R)	0.0625 (R)	1 (M)	0.5 (M)	0.25 (R)	0.25 (M)	2 (R)	0.015 (S)
CN18^*e*^	0.125 (R)	0.0625 (R)	1 (M)	1 (M)	1 (R)	0.125 (M)	2 (R)	0.015 (S)
CN19^*e*^	0.125 (R)	0.5 (R)	2 (M)	1 (M)	16 (R)	0.5 (M)	16 (R)	0.015 (S)
CN20^*e*^	0.125 (R)	>0.5 (R)	0.5 (M)	1 (M)	8 (R)	0.25 (M)	8 (R)	0.015 (S)
CN21^*e*^	0.125 (R)	>0.5 (R)	1 (M)	1 (M)	8 (R)	0.25 (M)	8 (R)	0.015 (S)
CN22^*e*^	0.125 (R)	>0.5 (R)	1 (M)	1 (M)	8 (R)	0.25 (M)	8 (R)	0.015 (S)
CN23^*e*^	0.125 (R)	0.5 (R)	2 (M)	1 (M)	16 (R)	0.5 (M)	16 (R)	0.015 (S)
CN24^*e*^	0.125 (R)	0.125 (R)	0.25 (S)	0.5 (M)	4 (R)	0.25 (M)	8 (R)	<0.015 (S)
CN25^*e*^	0.125 (R)	>0.5 (R)	1 (M)	1 (M)	4 (R)	0.125 (M)	8 (R)	0.015 (S)
CN26^*e*^	0.125 (R)	0.5 (R)	1 (M)	1 (M)	16 (R)	0.25 (M)	16 (R)	0.015 (S)
CN27^*e*^	0.125 (R)	0.5 (R)	1 (M)	1 (M)	16 (R)	0.5 (M)	16 (R)	0.015 (S)
CN28^*e*^	0.125 (R)	0.5 (R)	2 (M)	1 (M)	16 (R)	0.25 (M)	16 (R)	0.015 (S)
CN29^*e*^	0.125 (R)	0.5 (R)	2 (M)	1 (M)	16 (R)	0.5 (M)	16 (R)	0.015 (S)
CN30^*e*^	0.125 (R)	0.25 (R)	2 (M)	1 (M)	16 (R)	0.25 (M)	16 (R)	0.015 (S)
CN31^*e*^	0.125 (R)	0.5 (R)	2 (M)	1 (M)	16 (R)	0.5 (M)	16 (R)	0.015 (S)
								
CO_WT_^*f*^ (μg/ml)	<0.03 to ∼0.06	<0.002 to ∼0.03	0.5 to ∼4	0.25 to ∼1	0.03 to ∼0.12	<0.06 to ∼2	0.06 to ∼0.25	0.03 to >8

a R, resistant; M, moderate; S, susceptible; AMP, ampicillin; ENRO, enrofloxacin; ERY, erythromycin; FFN, florfenicol; FLUQ, flumequine; GEN, gentamicin; OXO, oxolinic acid; OXY, oxytetracycline.

b F. psychrophilum recovered from Liaoning province.

c F. psychrophilum recovered from Gansu province.

d F. psychrophilum recovered from Qinghai province.

e F. psychrophilum recovered from Heilongjiang province.

f The epidemiological cutoff values (CO_WT_) of F. psychrophilum to eight drugs were referenced by Van Vliet et al. (15).

Data from MIC analysis indicated that the 31 F. psychrophilum isolates exhibited reduced susceptibility to enrofloxacin (27/31, 87.1%), flumequine (29/31, 93.55%), and oxolinic acid (27/31, 87.1%) (Table 4). For ampicillin, the 31 F. psychrophilum isolates exhibited greater MIC variability, whereby 22/31 isolates (70.97%) showed reduced susceptibility, whereas 8/31 isolates (25.8%) were susceptible to ampicillin. Similar variability was observed in oxytetracycline MIC values, whereby 21/31 isolates (67.74%) showed reduced susceptibility. There were 4 isolates exhibiting reduced susceptibility to florfenicol, including CN01, CN03, CN06, and CN08. Of note, there were substantial differences in the susceptibility to ampicillin and oxytetracycline between CN01 to CN08 strains and CN09 to CN31 strains, which were isolated from different salmonid farms. CN01 to CN08 strains were resistant to oxytetracycline and highly susceptible to ampicillin, whereas CN09 too CN31 strains were highly susceptible to oxytetracycline.

## DISCUSSION

Flavobacterium psychrophilum is a bacterial fish pathogen of global significance, causing substantial economic losses in salmonid aquaculture on multiple continents (1, 6, 24, 25), yet published reports of its presence in China do not exist. In the current study, F. psychrophilum was confirmed as the causative agent of multiple BCWD outbreaks in several salmonid species raised in aquaculture facilities within four provinces in the northeastern and northwestern regions of China. Using a polyphasic approach, the biochemical, genetic, antimicrobial susceptibility, molecular serotype, and virulence profiles of representative isolates were determined, thereby generating data that are crucial not only for better understanding the global epidemiology of this pathogen but also for guiding the development of more effective and targeted means of preventing and controlling the losses caused by this pathogen in Chinese aquaculture.

Although the phenotype of F. psychrophilum isolates recovered from China varied somewhat, most matched descriptions from other regions of the world (1, 26). However, two isolates (CN20 and CN27) were able to grow in the nutrient-rich TSB medium, an attribute that has been rarely reported for a bacterium that grows most commonly on nutrient-poor media (27–30).

Isolates recovered from China fell into five distinct STs, with most (i.e., 23/31) belonging to three novel MLST singleton STs that are distinct from the >1,500 isolates that have been genotyped using the scheme of Nicolas et al. (11) and originated from five different continents (10, 12, 13, 31–36). Among the three newly discovered singletons, one (e.g., ST355) contained isolates that were recovered exclusively from one host species (e.g., rainbow trout), whereas ST356 and ST357 were recovered from two salmonid species belonging to two different genera (e.g., *Salvelinus* and *Oncorhynchus*) (Table 2). In this context, several studies have suggested that some F. psychrophilum MLST genotypes have strong predilections for particular host species, whereas others may be more host “generalists” (9–13, 34), a matter of importance toward better understanding the disease ecology and epidemiology of F. psychrophilum in China.

Genotyping via MLST also revealed that isolates belonging to CC-ST10, the largest and by far most globally widespread F. psychrophilum clonal complex that has a strong proclivity for rainbow trout (9–13, 34, 36), are also present in China (Table 2). In fact, the two CC-ST10 STs that were detected in China (e.g., ST12 and ST78) are single-locus variants (SLVs) of one of the largest currently recognized F. psychrophilum STs (e.g., ST10) (10). ST12 isolates had thus far been reported from South America and Europe (9, 11, 34), whereas ST78 had been found exclusively in North America (10, 13). Therefore, although ST12 and ST78 differ by a single AT, the fact that they were both repeatedly reported in regions of the world with a longer history of rainbow trout farming strongly suggests independent introduction of several sublineages of CC-ST10 in China, likely from different regions of the world. This should not seem surprising given the regular importation of rainbow trout eggs into China from Europe and the United States and the ability of this bacterium to be transmitted via infected eggs (37). Our observation is therefore consistent with the hypothesis that CC-ST10 was disseminated globally by international trade (38), pointing out the importance of inspection and quarantine for eggs and fry during transportation and aquaculture in salmonids.

Data from this study revealed that F. psychrophilum isolates from China thus far belong to at least two different molecular serotypes (e.g., type 0 and 1) that typically correspond to serotypes Fp^T^ and Fd, which were first identified in Denmark and recognized to be predominant in rainbow trout (17, 39). In the present study, all isolates retrieved from masou salmon belonged to type 0, whereas all isolates from rainbow trout and all but one isolate from brook trout belonged to type 1. Thus, the predilection of some F. psychrophilum serotypes to infect certain host species, as suggested by previous studies (19, 21, 40), also appears to hold true for isolates affecting salmonids in China. From a BCWD prevention perspective in relation to vaccine development, characterizing the antigenic profiles of the predominating F. psychrophilum strains behind the disease outbreaks is crucial (16). Indeed, recent studies in the United Kingdom have demonstrated the promise that whole-killed F. psychrophilum vaccine preparations (i.e., bacterins) hold when guided by serotyping data (41). In this context, data from this study will be useful as future research takes aim at developing vaccine preparations, which likely will need to be region- and/or fish species-specific in some cases. It is important to note, however, that the mPCR typing scheme of Rochat et al. (19) does not capture the entirety of F. psychrophilum antigenic diversity that is becoming increasingly apparent (42), but use of this assay is nevertheless an important step toward more targeted BCWD prevention.

Some serotypes (e.g., serotype Fd) are reportedly more virulent to rainbow trout under controlled laboratory conditions than others (43). Although the current study did not seek to compare the virulence of the two different mPCR types that were detected, CN06 and CN07 isolates (type 1) were virulent to juvenile rainbow trout (Fig. 5), in keeping with the previous reports. Interestingly, no type 2 isolates, which correspond to Th (described in 19), were found in China despite being widespread in Sweden, Denmark, and Finland (40); however, analysis of additional F. psychrophilum isolates recovered from Chinese aquaculture facilities is needed to fully assess the serological diversity that may be present in this vast country.

*In vitro* antimicrobial susceptibility assessments revealed that F. psychrophilum isolates from China exhibited reduced susceptibility to enrofloxacin, flumequine, and oxolinic acid and moderate susceptibility to gentamicin, erythromycin, and florfenicol, according to cutoff values previously established as relevant for F. psychrophilum (15). Of note, enrofloxacin is widely used to control BCWD epizootics in aquaculture facilities in China, which thus could be a factor in the observed increased MIC values for the analyzed isolates; however, further studies to test this hypothesis would be required. In a similar context, a reduced *in vitro* susceptibility to florfenicol, also approved for use against BCWD in China, was observed in several isolates, which has not been reported in F. psychrophilum isolates originating in the United States (15), United Kingdom (44), Turkey (45), or Chile (46). The similar findings were also reported in many countries and areas. Of 72 F. psychrophilum isolates from Ontario, Canada, 53% possessed high MIC values (≥2 μg/ml) to florfenicol (47). In Chile, 92.5% of the 40 F. psychrophilum isolates were resistant to florfenicol with the MIC values ranging from 4 μg/ml to 32 μg/ml (48). In Turkey, 50% of F. psychrophilum isolates had high MIC values (≥2 μg/ml) to florfenicol (49). Given these findings, it is of paramount importance that antibiotic treatments for BCWD be guided by antimicrobial susceptibility testing not only so that disease outbreaks are most efficaciously controlled but also to avoid the potential for perpetuating reduced antibiotic susceptibility of F. psychrophilum in China.

In the current study, the virulence of two CC-ST10 isolates (e.g., CN06 and CN07) belonging to two STs (e.g., ST12 and ST78) and originally recovered from rainbow trout from two Chinese provinces was assessed under laboratory conditions (Fig. 4). Indeed, both isolates induced severe and characteristic signs of BCWD in challenged fish and led to 90 to 100% mortality in the highest challenge doses. Although both isolates were recovered from disease outbreaks, where mortality ranged from 20 to 30% in affected groups, disease results from complex interactions between the host, the pathogen, and the environment (50), and it is also increasingly apparent that one or more etiological agent(s) can be involved in flavobacterial disease outbreaks (51, 52). In this context and given that little to no virulence data are available for F. psychrophilum strains affecting salmonids in China, data generated herein are noteworthy, as they not only show that the evaluated CC-ST10 isolates in China are virulent and capable of solely generating disease and mortality but also provide further evidence that CC-ST10 strains should be an important target in any current and future BCWD prevention and control initiatives.

In conclusion, data presented herein have elucidated the virulence, antimicrobial susceptibility, and molecular epidemiology of representative F. psychrophilum strains from China, which will serve as a platform from which both broad and targeted BCWD prevention and control strategies can be developed and deployed within Chinese aquaculture facilities. Future studies will focus on more thoroughly addressing F. psychrophilum prevalence, transmission, and infection reservoirs across China.

## MATERIALS AND METHODS

### Fish and sampling.

Representative affected fish farmed in Heilongjiang and Liaoning provinces were transported alive to the Heilongjiang River Fisheries Research Institute-Aquatic Animal Health Laboratory (Harbin, Heilongjiang Province), euthanized by sodium bicarbonate buffered tricaine methanesulfonate (MS-222) overdose, and then grossly examined externally and internally. For sampling from the affected farms in the Gansu and Qinghai Provinces, fish were euthanized and bacteriological assessments (see below) were initiated on site.

### Bacterial isolates and growth conditions.

For primary bacterial isolation, tissue from the kidney, spleen, and/or skin ulcers of juvenile fish were streaked directly onto modified TYES (tryptone yeast extract salts medium: 0.4% tryptone, 0.05% yeast extract, 0.02% anhydrous calcium chloride, 0.05% magnesium sulfated heptahydrate, 12 g/liter agar [pH 7.2]) agar plates (28) using sterile disposable 10-μl loops and then incubated aerobically at 15°C for up to 5 days. The ability of bacterial isolates to grow in tryptic soy broth (TSB) was also evaluated by incubation of the bacterium at 15°C, 120 rpm for 72 h. For cryopreservation, bacterial isolates were grown in TYES broth at 15°C for 48 h, glycerol was added (20% vol/vol), and then cultures were immediately frozen at −80°C. Escherichia coli ATCC 25922 and Aeromonas salmonicida subsp. *salmonicida* ATCC 33658 were used as the quality control strains for antimicrobial susceptibility test (53).

### Phenotypic characterization and scanning electron microscopy.

Bacterial isolates were revived from cryostock into TYES broth and incubated for 72 to 96 h at 15°C, at which time bacterial cells were subcultured onto fresh TYES agar to verify purity. Next, isolates were examined using multiple phenotypic tests, including Gram-staining, cytochrome oxidase and catalase activities, presence of flexirubin-type pigment, colony and cell morphology, and gliding motility by the hanging-drop technique (54, 55). Gelatin hydrolysis was tested by stabbing isolates into test tubes containing 0.5 ml 2× TYES supplemented with 4% gelatin. Commercial biochemical test tubes (Hopebio, Qingdao, China) were used to examine utilization of glucose, fructose, galactose, mannose, and glycerol, production of hydrogen sulfide, and hydrolysis of esculin and starch at 15°C for 48 to 72 h. Yellow-pigmented bacterial isolates originally recovered on TYES that were Gram-negative and rod-shaped, produced a flexirubin-type pigment, and were catalase and oxidase positive were selected for further molecular analysis. Morphology observation of a representative isolate was performed using negative-stain scanning electron microscopy (SEM) analysis following the procedure from our previous study (56).

### Molecular characterization.

Bacterial isolates were revived as described above. Chromosomal DNA was then extracted using the bacteria genomic DNA extraction kit (Tiangen, China) according to the manufacturer’s protocol. The concentration of DNA was measured using an Agilent 2100 Bioanalyzer (Agilent Technologies, CA, USA).

Bacterial identity was confirmed using 16S rRNA amplification and gene sequencing, whereby PCR was conducted using the universal primers 27F (5′-AGAGTTTGATCMTGGCTCAG-3′) and 1492R (5′-GGTTACCTTGTTACGACTT-3′), giving a final product of ∼1,400 bp (57). The 50 μl PCR reaction for each sample contained aliquots of each primer at a final concentration of 150 nM each, 25 μl of 2× GoTaq green master mix (Promega), and 40 ng of DNA template, with DNase-free water comprising the remainder of the mixture. The amplification program was as follows: initial denaturation at 95°C for 5 min, followed by 30 cycles of denaturation at 94°C for 1 min, annealing at 55°C for 1 min, extension at 72°C for 2 min, and a final extension at 72°C for 10 min. The amplicons were visualized on a 1.2% agarose gel (58). The amplicons were purified using the QIAquick PCR purification kit (Qiagen, Germany) and then sequenced with 27F and 1492R primers by Comate Bioscience Co. Ltd. (Changchun, China). Contigs were assembled using BioEdit software and analyzed against a database of quality-controlled 16S rRNA sequences (EZBioCloud; https://www.ezbiocloud.net/), where comparisons in percent similarity were made (59).

For MLST, partial sequences of seven housekeeping genes (*trpB*, *gyrB*, *dnaK*, *fumC*, *murG*, *tuf*, and *atpA*) selected previously (11) were PCR amplified (13) and bidirectionally Sanger-sequenced using the F. psychrophilum MLST sequencing primers as described previously (12). All chromatograms were verified for quality using an in-house script (INRAE) prior to AT and ST assignment. AT profiles were compared to those of all F. psychrophilum isolates in the publicly available PubMLST database (https://pubmlst.org/fpsychrophilum/) (60), accessed in March 2021. A single-linkage hierarchical clustering tree based on a pairwise distance corresponding to the number of divergent ATs between STs was built with “hclust” R function and drawn with “plot.phylo” (61). goeBURST (http://phyloviz.net/goebursthttp://phyloviz.net/goeburst) (62) was used to reconstruct the relationships between STs to identify if they belonged to a clonal complex (CC; i.e., a group of STs that share identical allele types at six out of seven loci) or constituted a singleton ST (i.e., a ST that is not part of a CC).

### Characterization of molecular serotype.

All confirmed F. psychrophilum isolates recovered from China were analyzed to determine the molecular serotype using the multiplex PCR (mPCR)-based serotyping scheme (19). The mPCR reactions were performed using 2 μl of purified genomic DNA, 25 μl of 2× platinum multiplex PCR master mix (Thermo Fisher Scientific), and 10 μM aliquots of each primer in a 50-μl final reaction volume. The mPCR amplification mix was heated at 95°C for 5 min, followed by 30 cycles of 95°C for 30 s, 52°C for 30 s, 72°C for 60 s, and a final extension at 72°C for 10 min. DL2000 marker was used to estimate the fragment size, and serotypes were classified into type 0 (188 bp), type 1 (188 bp and 549 bp), type 2 (188 bp and 841 bp), and type 3 (188 bp and 361 bp) (19).

### Antimicrobial susceptibility testing.

The MICs for ampicillin (AMP), enrofloxacin (ENRO), erythromycin (ERY), florfenicol (FFN), flumequine (FLUQ), gentamicin (GEN), oxytetracycline (OXY), and oxolinic acid (OXO) against F. psychrophilum isolates were assessed using the broth microdilution protocol recommended for F. psychrophilum in the CLSI guideline VET04-A2. The antibiotics were purchased from Macklin Biochemical Co., Ltd. (Shanghai, China). All broth dilution preparations were performed according to CLSI under aseptic conditions (53). Quality control strains ATCC 25922 of E. coli and ATCC 33658 of A. salmonicida subsp. salmonicida were prepared alongside each group of F. psychrophilum isolates as recommended and detailed in CLSI. The antimicrobial susceptibility patterns of the isolates were analyzed based on the epidemiological cutoff values (15).

### *In vivo* virulence experiments.

The *in vivo* virulence of two F. psychrophilum isolates, CN06 and CN07, was assessed via intramuscular injection in juvenile rainbow trout. Prior to virulence experiments, healthy rainbow trout (mean body weight of 25 g) were kept under laboratory conditions in flowthrough tanks at approximately 12°C with continuous aeration and fed twice a day at 1.2% of body weight with commercial fish feed (Aller Aqua, Qingdao, China). Prior to the experimental challenge, a subset of rainbow trout was microscopically and bacteriologically examined to verify absence of F. psychrophilum infection.

To prepare F. psychrophilum for challenge, CN06 and CN07 were revived from cryostock onto TYES agar, incubated for 3 to 4 days at 15°C, and then inoculated into TYES broth, which was incubated statically for 3 days at 15°C. Bacteria were then harvested via centrifugation and resuspended in sterile phosphate-buffered saline (PBS), and colony counts were performed via serial 10-fold dilutions and plate counts.

Twenty rainbow trout (*n = *10 fish per tank; in duplicate) were anesthetized in MS-222 and intramuscularly injected with CN06 and CN07 bacterial suspension at the base of the adipose fin. For CN06 isolate, each group was injected intramuscularly (i.m.) with 4.5 × 10^8^, 4.5 × 10^7^, 4.5 × 10^6^, 4.5 × 10^5^, and 4.5 × 10^4^ CFU of F. psychrophilum suspended in 100 μl sterile PBS buffer per fish. For CN07 isolate, each group was injected i.m. with 9 × 10^7^, 9 × 10^6^, 9 × 10^5^, 9 × 10^4^, and 9 × 10^3^ CFU of F. psychrophilum suspended in 100 μl sterile PBS buffer per fish. Control fish (*n *= 10 fish per tank; in duplicate) were mock infected with 100 μl of sterile PBS buffer. Following the challenge, fish were immediately transferred into aerated tanks with flowthrough water at approximately 12°C and fed twice a day at 1.5% of body weight with commercial feed. Fish were monitored daily for 14 days, and the cumulative percent mortality was presented by dividing dead fish numbers by 10 for each challenge dose. Mortalities were necropsied, clinically examined, and bacteriologically analyzed. Moribund fish and survivors were euthanized via an overdose of MS-222 and examined identically. The median lethal dose (LD_50_) of each isolate in juvenile rainbow trout was calculated using trimmed Spearman-Karber’s method (63). To confirm the presence of F. psychrophilum in different host tissues (e.g., spleen, liver, kidney, and muscle), the F. psychrophilum-specific qPCR assay was used (64).

### Ethics statement.

The Committee of the Ethics on Animal Care and Experiments at Heilongjiang River Fisheries Research Institute of Chinese Academy of Fishery Sciences approved this study, which was consistent with the Guidelines of European Directive 2010/2063/EU for the protection of experimental animals.

### Data availability.

The 16S rRNA gene sequences of 31 F. psychrophilum isolates are available in the GenBank of NCBI under accession numbers MN653377 to MN653384 and MT249231 to MT249253. For MLST, sequences and background information on isolates are available in PubMLST (https://pubmlst.org/fpsychrophilum/) and Table 2.
