# Supplementary material for: Phenotypic and Genetic Characterization of Flavobacterium psychrophilum Recovered from Diseased Salmonids in China
Source: Microbiol Spectr. 2021 Sep 15;9(2):e00330-21. doi: 10.1128/Spectrum.00330-21 (PMC8557942; doi:10.1128/Spectrum.00330-21)
Supplement: SUPPLEMENTAL FILE 1 — Supplemental material. Download SPECTRUM00330-21_Supp_1.pdf, PDF file, 0.1 MB [file spectrum00330-21_supp_1.pdf]

TABLE S1 MLST profiles of *F. psychrophilum* presented in this study. Data is arranged by sequence type (ST).

ST	ATs						
	atpA	*dnaK*	*fumC*	*gyrB*	*murG*	*trpB*	*tuf*
ST10	2	2	2	8	2	2	2
ST12	2	2	2	8	2	2	7
ST13	4	6	5	7	6	4	8
ST78	2	2	2	8	2	2	41
ST256	59	4	2	28	25	1	25
ST258	14	26	5	18	47	42	8
ST275	2	2	2	8	2	2	62
ST286	64	9	5	76	1	1	65
ST341	2	2	2	8	2	11	62
ST342	2	2	2	8	2	2	73
ST343	76	10	3	12	3	1	17
ST344	2	10	3	12	3	1	17
ST345	57	7	3	82	3	21	5
ST346	11	36	3	72	13	18	6
ST347	57	15	10	69	50	4	12
ST349	1	9	5	76	1	1	74
ST350	7	8	3	9	12	6	5
ST351	77	8	10	90	16	48	75
ST352	14	9	6	49	35	48	50
ST353	14	7	18	91	25	15	76
ST354	11	15	3	2	13	4	3
ST355	8	8	19	40	10	49	7
ST356	23	26	5	4	18	4	47
ST357	23	37	5	2	34	50	3
