# Supplementary material for: Phenotypic and Genetic Characterization of Flavobacterium psychrophilum Recovered from Diseased Salmonids in China
Source: Microbiol Spectr. 2021 Sep 15;9(2):e00330-21. doi: 10.1128/Spectrum.00330-21 (PMC8557942; doi:10.1128/Spectrum.00330-21)
Supplement: SUPPLEMENTAL FILE 2 — Supplemental material. Download SPECTRUM00330-21_Supp_2.pdf, PDF file, 0.1 MB [file spectrum00330-21_supp_2.pdf]

TABLE S2 Complete information of 37 North American *F. psychrophilum* isolates presented in this study including year of isolation, location of isolation, host species, tissue of isolation, host life stage and genotype. Data is arranged by clonal complex (CC) then sequence type (ST).

Isolate ID	Year of isolation	Location of isolation	Species	Isolation tissue	Wild/feral or captive	Life stage	ST[d]	CC
US453	2018	Michigan	*O. kisutch*	Kidney	Wild/feral	Adult[b]	ST13	CC-ST9
US458	2019	Michigan	*O. kisutch*	Kidney	Captive	Juvenile[b]	ST13	CC-ST9
US456	2019	New York	*O. mykiss*	Unknown	Captive	Juvenile[a]	ST10	CC-ST10
US457	2019	New York	*O. mykiss*	Unknown	Captive	Juvenile[a]	ST10	CC-ST10
US463	2019	Michigan	*O. mykiss*	Kidney	Captive	Juvenile[b]	ST10	CC-ST10
US459	Unknown	Wyoming	*Unknown*	Unknown	Unknown	Unknown[c]	ST78	CC-ST10
US464	2019	Michigan	*O. mykiss*	Kidney	Captive	Juvenile[b]	ST275	CC-ST10
US398	2018	Michigan	*O. mykiss*	Kidney	Wild/feral	Adult[b]	ST341	CC-ST10
US399	2018	Michigan	*O. mykiss*	Kidney	Wild/feral	Adult[b]	ST341	CC-ST10
US400	2018	Michigan	*O. mykiss*	Kidney	Wild/feral	Adult[b]	ST342	CC-ST10
US445	2018	Wisconsin	*O. kisutch*	Unknown	Unknown	Unknown[c]	ST256	CC-ST256
US455	2019	Michigan	*O. mykiss*	Caudal fin	Captive	Juvenile[a]	ST354	CC-ST287
US401	2018	New Jersey	*O. mykiss*	Kidney	Captive	Juvenile[a]	ST343	CC-ST343
US402	2018	New Jersey	*O. mykiss*	Kidney	Captive	Juvenile[a]	ST343	CC-ST343
US403	2018	New Jersey	*O. mykiss*	Kidney	Captive	Juvenile[a]	ST343	CC-ST343
US404	2018	New Jersey	*O. mykiss*	Kidney	Captive	Juvenile[a]	ST344	CC-ST343
US414	2018	Michigan	*O. tshawytscha*	Kidney	Wild/feral	Adult[b]	ST347	CC-ST347
US 415	2018	Michigan	*O. tshawytscha*	Kidney	Wild/feral	Adult[b]	ST347	CC-ST347
US409	2018	Michigan	*O. tshawytscha*	Kidney	Wild/feral	Adult[b]	ST345	Singleton
US410	2018	Michigan	*O. tshawytscha*	Kidney	Wild/feral	Adult[b]	ST345	Singleton
US411	2018	Michigan	*O. tshawytscha*	Kidney	Wild/feral	Adult[b]	ST345	Singleton
US412	2018	Michigan	*O. tshawytscha*	Kidney	Wild/feral	Adult[b]	ST345	Singleton
US413	2018	Michigan	*O. tshawytscha*	Kidney	Wild/feral	Adult[b]	ST346	Singleton
US439	2018	Michigan	*O. mykiss*	Kidney	Captive	Juvenile[b]	ST349	Singleton

US442	2018	Michigan	*S. salar*	Kidney	Wild/feral	Adult[b]	ST350	Singleton
US443	2019	Michigan	*O. kisutch*	Kidney	Captive	Juvenile[b]	ST350	Singleton
US444	2018	Wisconsin	*S. fontinalis*	Unknown	Unknown	Unknown[c]	ST351	Singleton
US446	2018	Wisconsin	*O. kisutch*	Unknown	Unknown	Unknown[c]	ST258	Singleton
US447	2018	Wisconsin	*O. kisutch*	Unknown	Unknown	Unknown[c]	ST258	Singleton
US448	2019	Wisconsin	*O. kisutch*	Unknown	Unknown	Unknown[c]	ST258	Singleton
US454	2018	Michigan	*O. kisutch*	Kidney	Wild/feral	Adult[b]	ST258	Singleton
US461	2019	Michigan	*S. trutta*	Gill	Captive	Juvenile[a]	ST286	Singleton
US462	2019	Michigan	*S. trutta*	Eye	Captive	Juvenile[a]	ST286	Singleton
US452	2018	Michigan	*O. kisutch*	Kidney	Wild/feral	Adult[b]	ST350	Singleton
US449	2018	Wisconsin	*S. trutta*	Unknown	Unknown	Unknown[c]	ST352	Singleton
US450	2019	Michigan	*S. namaycush*	Dorsal fin	Captive	Juvenile[a]	ST353	Singleton
US451	2019	Michigan	*S. namaycush*	Dorsal fin	Captive	Juvenile[a]	ST353	Singleton

[a] Isolate was recovered from a fish experiencing BCWD.

[b] Isolate was recovered from an apparently healthy fish.

[c] Fish disease status was unknown.

[d] STs in italics were newly discovered in this study.
